# The complete chloroplast genome sequence of *Passiflora xishuangbannaensis* (Passifloraceae), a vine endemic to Yunnan, China

**DOI:** 10.1080/23802359.2021.1928562

**Published:** 2021-05-27

**Authors:** Chunhui Hao, Fuchuan Wu

**Affiliations:** a Xishuangbanna Tropical Botanical Garden, Chinese Academy of Sciences, Mengla, Yunnan, China; b University of Chinese Academy of Sciences, Beijing, China

**Keywords:** Chloroplast, *Passiflora xishuangbannaensis*, phylogenetic analysis

## Abstract

*Passiflora xishuangbannaensis*, *Passiflora* subgenus *Decaloba*, is a very rare endemic to the Yunnan, China. Here we report and characterize the complete chloroplast (cp) genome sequence of *P. xishuangbannaensis* to provide genomic resources useful for promoting its conservation and systematics. The complete genome is 135,742 bp in length and the overall GC content is 37.1%. The cp genome sequence has a typical quadripartite structure, comprising two inverted repeats (IRs: 20,604 bp) regions, which are separated by a small single-copy (SSC: 13,159 bp) region and a large single-copy (LSC: 81,375 bp) region. Moreover, a total of 122 functional genes were annotated, including 77 protein-coding genes, 37 tRNA genes, and 8 rRNA genes. The phylogenetic analysis recovered *P. xishuangbannaensis* as a member of subgenus *Decaloba*.

*Passiflora xishuangbannaensis* Krosnick (Passifloraceae) is a vine endemic to the southern Yunnan Province of China. Its native range is restricted to Xishuangbanna Prefecture, Yunnan Province, China (Krosnick 2005). The species is classified to the genus *Passiflora*, subgenus *Decaloba*, supersection *Disemma* (Krosnick et al. 2013). *Passiflora xishuangbannaensis* was recently described and was said to differ from its most closely related species, *P. altebilobata* Hemsley, by being glabrous, having less flowers at each node, deeply bilobed leaves, and a v-shaped arrangement of numerous abaxial laminar nectaries (Krosnick 2005). Following several field surveys, very few individual have been recorded, it is therefore considered to be very rare. It is mainly distributed in the shaded areas along and near streams in primary forests. Currently, the habitat is greatly affected by road construction and farming. It is urgent to design an effective conservation strategy to protect this species. In this study, we report the complete chloroplast genome sequence of *P. xishuangbannaensis* to provide genomic resources useful for promoting its conservation, biodiversity, and systematic research.

Fresh leaf samples of *P. xishuangbannaensis* were collected from Xishuangbanna Tropical Botanical Garden (XTBG), Chinese Academy of Sciences, Yunnan Province, China (N21°41′, E101°25′). A voucher specimen was deposited at the Herbarium of XTBG (http://hitbc.xtbg.ac.cn, Jianwu Li, ljw@xtbg.org.cn) under the voucher number BN-01. Total genomic DNA was extracted using the CTAB method (Doyle and Dickson 1987). High-throughput sequencing of the plastid genomes was performed using the Illumina Novaseq 6000. A total of 6.07 GB raw reads were generated. After filtering using Fastp (Chen et al. 2018), 6.05 GB of clean reads data were obtained. GetOrganelle toolkit (Jin et al. 2020) were employed to assemble the chloroplast genome. The plastid genome sequences of *P. rufa* (NC043817.1), *P. jatunsachensis* (NC043813.1), and *P. microstipula* (NC043827.1) were used as references to annotate the assembled chloroplast genome by the web server GeSeq (https://chlorobox.mpimp-golm.mpg.de/geseq.html, Michael et al. 2017). Geneious Primer v2021.0.3 was used to check the accuracy of the assembly. The raw sequencing reads used in this study have been deposited in the Sequence Read Archive (SRA) with the accession number SRX10188724, the annotated chloroplast genome sequence was deposited in GenBank under the accession number MW540528.

The complete cp genome sequence of *P. xishuangbannaensis* is 135,742 bp in length. A total of 122 genes were annotated, including 77 protein-coding genes, 37 tRNA genes, and 8 rRNA genes. It has a typical quadripartite structure containing two inverted repeats (IRs) regions each 20,604 bp in length, separated by a small single-copy (SSC) region of 13,159 bp in length and a large single-copy (LSC) region of 81,375 bp in length. The overall GC content is 37.1%, and the A, T, C, G base composition of the genome is 31, 31.9, 18.9, and 18.2%, respectively.

To better understand the phylogenetic position of *P. xishuangbannaensis*, a maximum likelihood (ML) phylogenetic tree was inferred based on 72 plastid encoded protein genes, of which 24 species from *Passiflora* and one species of *Populus* served as the outgroup (Figure 1). The sequence alignment was conducted using the MAFFT (Katoh and Standley 2013) plugin in Geneious. The best-fit nucleotide substitution model of ML analysis were calculated using Models function in MAGE-X 1.6.12. The phylogenetic tree using MAGE-X with 1,000 bootstrap replicates under the best-fit model GTR + G+I. The phylogenetic analyses resolved *P. xishuangbannaensis* in a clade in the subgenus *Decaloba* (Figure 1). The result is consistent with previous publication (Krosnick et al. 2013), based on the strict consensus of ITS data, *P. xishuangbannaensis* was strongly supportde as a member of *Passiflora* subgenus *Dacoloba*. The complete cp genome of *P. xishuangbannaensis* contributes to the development of conservation strategies for this rare species, as well as for further phylogenetic studies of the Passifloraceae.

**Figure 1.**
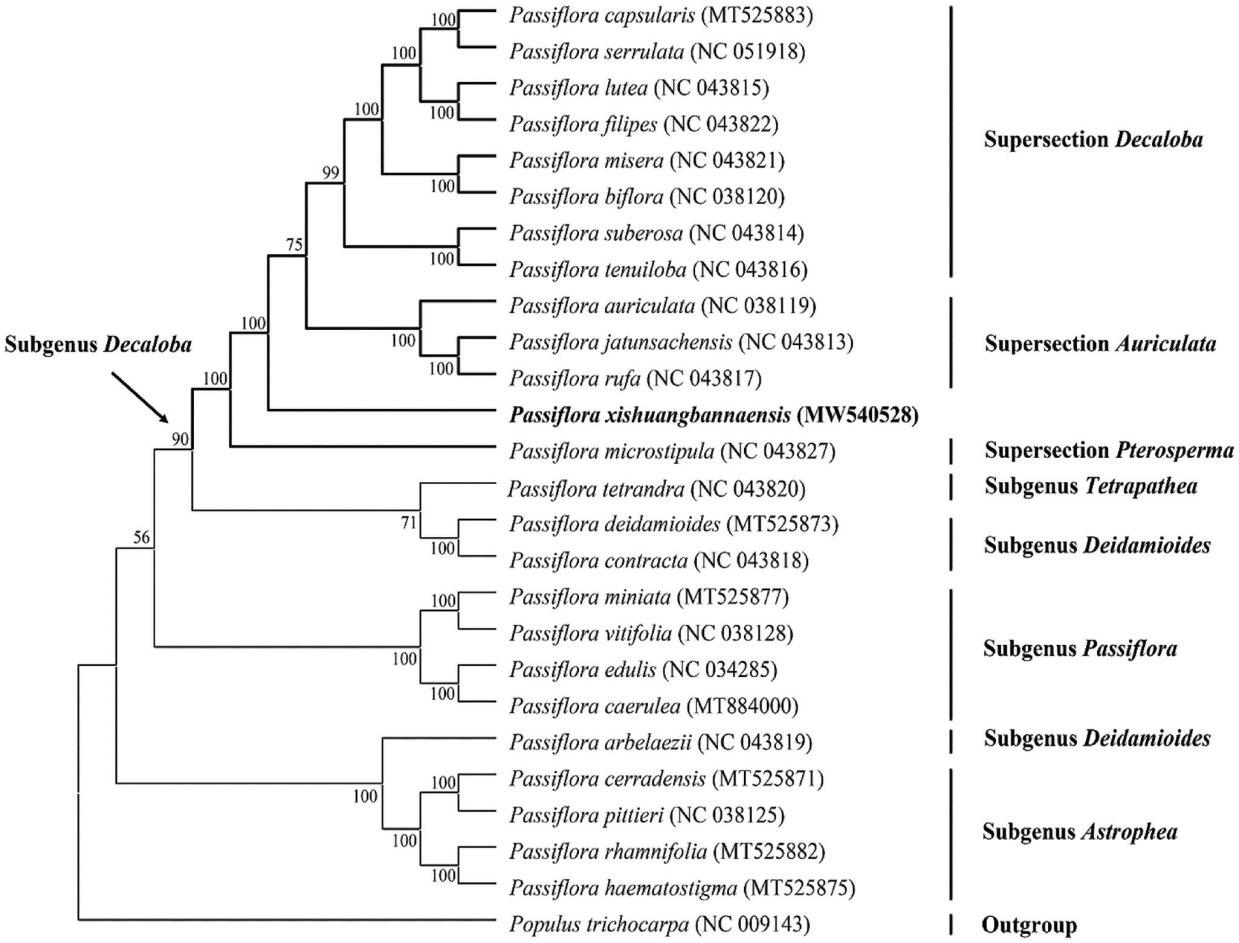
Phylogenetic position of *Passiflora xishuangbannaensis* inferred by the maximum-likelihood (ML) analysis based on 72 protein-coding genes from chloroplast genome using 1000 bootstrap replicates. Numbers above/below the branch lines represent ML bootstrap values based on 1,000 replicates.

## Data Availability

The genome sequence data that support the findings of this study are openly available in GenBank of NCBI at [https://www.ncbi.nlm.nih.gov] (https://www.ncbi.nlm.nih.gov/) under the accession no. MW540528. The associated BioProject, SRA, and BioSample numbers are PRJNA705404, SRX10188724, and SAMN18087741, respectively.
